# *Mycobacterium tuberculosis* infection in cattle from the Eastern Cape Province of South Africa

**DOI:** 10.1186/s12917-017-1220-3

**Published:** 2017-10-10

**Authors:** Tiny Motlatso Hlokwe, Halima Said, Nomakorinte Gcebe

**Affiliations:** 1 Tuberculosis Laboratory, Diagnostic Services Programme, ARC-Onderstepoort Veterinary Research, Private Bag X05, Onderstepoort, Pretoria, 0110 South Africa; 2 Division of the National Health Laboratory Services, Centre for Tuberculosis, National Institute for Communicable Diseases, Private Bag X4, Sandringham, Johannesburg, South Africa

**Keywords:** *Mycobacterium tuberculosis*, Cattle (*Bos taurus*), Genotyping, Zoonosis

## Abstract

**Background:**

*Mycobacterium tuberculosis* is the main causative agent of tuberculosis (TB) in human and *Mycobacterium bovis* commonly causes tuberculosis in animals. Transmission of tuberculosis caused by both pathogens can occur from human to animals and vice versa.

**Results:**

In the current study, *M. tuberculosis,* as confirmed by polymerase chain reaction (PCR) using primers targeting 3 regions of difference (RD4, RD9 and RD12) on the genomes, was isolated from cattle originating from two epidemiologically unrelated farms in the Eastern Cape (E.C) Province of South Africa. Although the isolates were genotyped with variable number of tandem repeat (VNTR) typing, no detailed epidemiological investigation was carried out on the respective farms to unequivocally confirm or link humans as sources of TB transmission to cattle, a move that would have embraced the ‘One Health’ concept. In addition, strain comparison with human *M. tuberculosis* in the database from the E.C Province and other provinces in the country did not reveal any match.

**Conclusions:**

This is the first report of cases of *M. tuberculosis* infection in cattle in South Africa**.** The VNTR profiles of the *M. tuberculosis* strains identified in the current study will form the basis for creating *M. tuberculosis* VNTR database for animals including cattle for future epidemiological studies. Our findings however, call for urgent reinforcement of collaborative efforts between the veterinary and the public health services of the country.

## Background

*Mycobacterium tuberculosis* and *Mycobacterium bovis* are the most commonly encountered members of the *Mycobacterium tuberculosis* complex (MTBC) species worldwide. The former is the main causative agent of TB in humans, and the latter is responsible for the disease mainly in animals [1, 2]. Transmission of tuberculosis caused by both pathogens can occur from human to animals and vice versa [3–6]. *M. tuberculosis* does not appear to have an indigenous animal maintenance host and the animals that become infected represent most probably incidental host [7]. Not much is known about the prevalence of *M. tuberculosis* in its spill over hosts. *M. tuberculosis* infected animals including cattle react positively when subjected to tuberculin skin testing (TST); however, the infection seems to vanish rather quickly and does not normally lead to a progressive disease [7–9]. *M. tuberculosis* infections in cattle is known to occur mostly in countries with the highest incidences of human tuberculosis in Africa and Asia [10]. The prevalence of *M. tuberculosis* infections in cattle in isolated studies was reported to be 6.2% in Algeria [11], 7.4% in Sudan [12], 7% and approximately 27% in different studies conducted in Ethiopia [13, 14]. It should however, be noted that these prevalence was only for isolated studies and do not reflect the individual country‘s true prevalence. In a study conducted in Nigeria, *M. tuberculosis* was isolated from cattle that reacted positive to caudal fold intradermal tuberculin test (bovine tuberculin test). The cattle attendant was also diagnosed with active pulmonary tuberculosis. Although genotyping of the isolates was not conducted, the cattle attendant was suggested to be the most probable source of *M. tuberculosis* in cattle [15].

In South Africa, TB in cattle is known to be caused by *M. bovis.* Control strategies have already been in place since 1969, and reduced the disease prevalence to less than 1%, although sporadic outbreaks still occur [16, 17]. On the contrary, our country was amongst the six countries with the highest incidence of human TB, with the estimated incidence of 450,000 cases of active disease reported in 2013 [18]. According to the South African government, 73% of these TB patients were co-infected with HIV (Human Immunodeficiency Virus). Multidrug resistant (MDR) and extensively drug-resistant (XDR) strains of *M. tuberculosis* occur, with some strains linked to high mortality rate [19] and some suggested to be hyper virulent with greater ability to evade host defences [20]. Investigators in several countries have traced the sources of *M. tuberculosis* on cattle farms as humans with active TB [10, 14, 21]. Tracing of sources of infections is mainly achieved by conducting molecular typing techniques to establish the epidemiological relatedness of the strains from both cattle and humans [6, 21]. Transmission to cattle usually occurs via sputum but rarely urine and faeces of infected individuals [22].

We report in the current study, the infection of cattle with *M. tuberculosis* in two cases from different farms in the Eastern Cape (E.C) Province of South Africa (S.A). Our work is first to report *M. tuberculosis* infections in cattle in South Africa. *M. tuberculosis* is zoonotic and some strains are known to be highly virulent. As such, infection in cattle is a public health concern since there is a risk of spill back to humans, especially in immunocompromised individuals. We found it imperative to conduct molecular characterization of the cattle strains in an attempt to elucidate sources of infection by comparison with those in the local human data base. The genotyping profiles generated in the study will form the basis for creation of a database for future epidemiological studies of *M. tuberculosis* infections in animals including cattle.

## Results

**Fig. 1:**
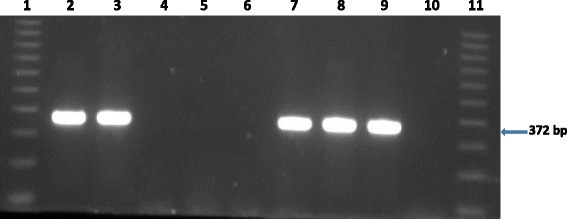
Gel electrophoresis results of the Polymerase Chain Reaction (PCR) products for the identification of *Mycobacterium tuberculosis* complex bacteria. Lane 1 and 11, molecular weight marker (100 bp ladder). Lane 2, TB 7000; lane 3, TB 7046A; lane 4, TB 6985A; lane 5, TB 6985B; lane 6, TB 7047, lane 7–8, *Mycobacterium bovis* in house controls; lane 9, *Mycobacterium tuberculosis* H37Rv control; lane 10, H_2_O control. bp = base pairs

**Fig. 2:**
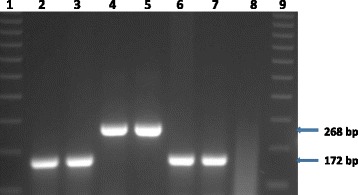
Gel electrophoresis results of the RD4 Polymerase Chain Reaction (PCR) products for the identification of *Mycobacterium tuberculosis* Lane 1 and 9, molecular weight marker (100 bp ladder). Lane 2, TB 7000; lane 3, TB 7046A; lane 4–5, *Mycobacterium bovis* in house controls; lane 6, *Mycobacterium tuberculosis* in house control; lane 7, *Mycobacterium tuberculosis* H37Rv control; lane 8, H_2_O control. bp = base pairs

**Fig. 3:**
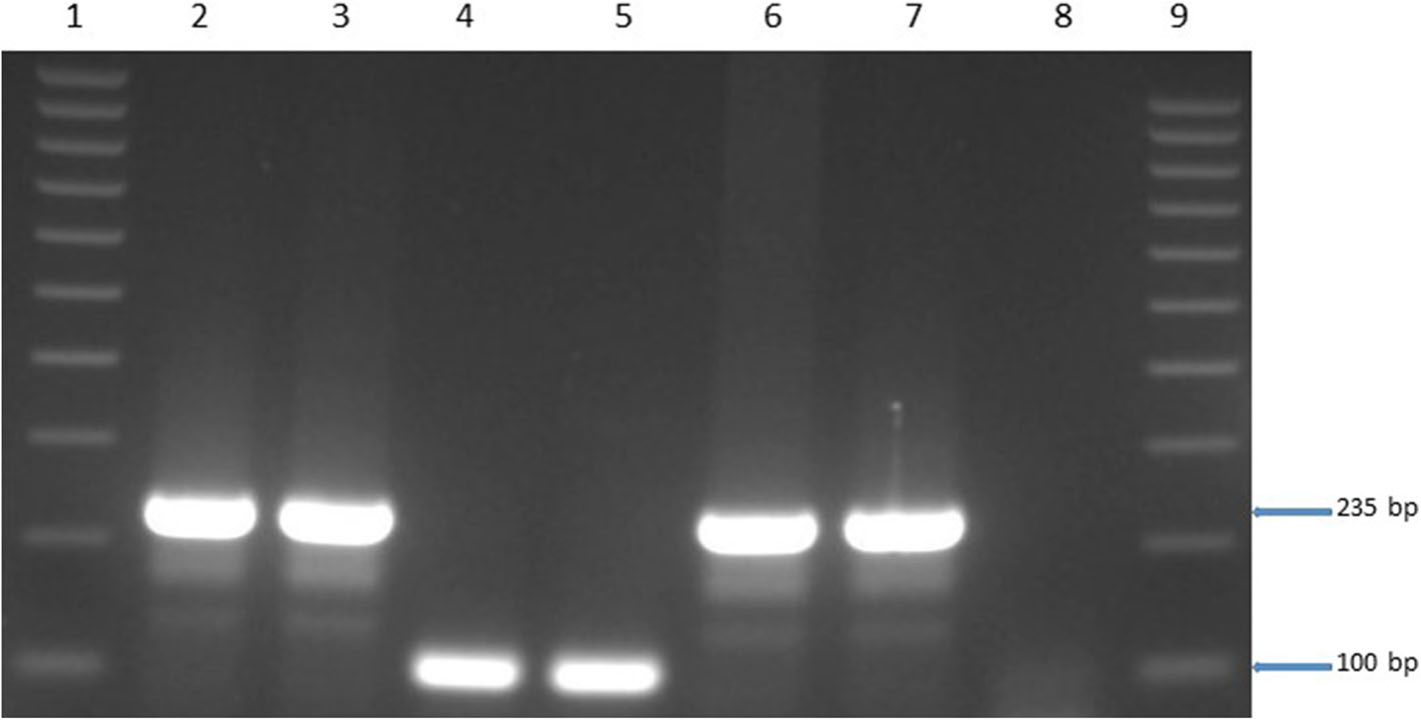
Gel electrophoresis results of the RD9 Polymerase Chain Reaction (PCR) products for the identification of *Mycobacterium tuberculosis* Lane 1 and 9, molecular weight marker (100 bp ladder). Lane 2, TB 7000; lane 3, TB 7046A; lane 4–5, *Mycobacterium bovis* in house controls; lane 6, *Mycobacterium tuberculosis* in house control; lane 7, *Mycobacterium tuberculosis* H37Rv control; lane 8, H_2_O control. bp = base pairs

**Fig. 4:**
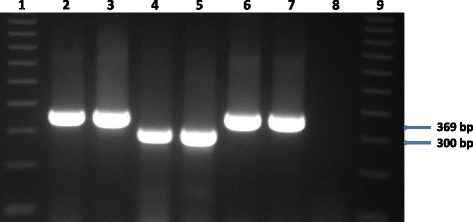
Gel electrophoresis results of the RD12 polymerase chain reaction (PCR) products for the identification of *Mycobacterium tuberculosis* Lane 1 and 9, molecular weight marker (100 bp ladder). Lane 2, TB 7000; lane 3, TB 7046A; lane 4–5, *Mycobacterium bovis* in house controls; lane 6, *Mycobacterium tuberculosis* in house control; lane 7, *Mycobacterium tuberculosis* H37Rv control; lane 8, H_2_O control. bp = base pairs

Acid-fast organisms were isolated from a lung sample (TB 7000; animal ID 25545); and prescapsular (TB 6985A) and mesenteric (TB 6985B) lymph nodes (animal ID 83750233) from cattle originating from farm 1. On farm 2, isolation was made from a prescapsular lymph node (TB 7046A; animal ID 84750367) and from a mediastinal lymph node (TB 7047; animal ID 23738). No visible tuberculous lesions were observed in any of these samples (Table 1). Two mycobacterial isolates (i.e. TB 7000 and TB 7046A), one from each farm, were confirmed as members of the *Mycobacterium tuberculosis* complex (MTBC) species by PCR amplification of the MTBC specific 372 bp product. No PCR product was observed for the other acid-fast bacteria. Deletion analysis PCR using primers targeting individual regions of differences RD4 (172 bp product), RD9 (235 bp product) and RD12 (369 bp product) identified the isolates as *M. tuberculosis* (Figs. 1, 2, 3 and 4). The other three isolates that tested negative on the MPB70 PCR were identified as *Mycobacterium nonchromogenicum* (TB 6985A and TB 6985B) and a non-tuberculous mycobacterium (NTM) species closely related to *Mycobacterium moriokaense* by 16S rDNA polymerase chain reaction (PCR) and sequence analysis. Information regarding all samples cultured and the *Mycobacterium* species isolated is presented in Table 1.

**Table 1:** Animal identification, culture results and identification of *Mycobacterium* species isolated from tissue samples of cattle originating from two different farms in the Eastern Cape Province of South Africa

Laboratory identification	Animal identification	Sample type	Laboratory macroscopic examination of lesions	Culture results	Mycobacterium species identification	Farm
TB 6982	83750230	Mesenteric lymph node	Absent	Negative	N/A	Farm 1
TB 6983	83750231	Prescapsular lymph node	Absent	Negative	N/A	Farm 1
TB 6984	83750232	Mesenteric lymph node	Absent	Negative	N/A	Farm 1
TB 6985A	83750233	Prescapsular lymph node	Absent	Positive	*M. nonchromogenicum*	Farm 1
TB 6985B	83750233	Mesenteric lymph node	Absent	Positive	*M. nonchromogenicum*	Farm 1
TB 6986	83750234	Bronchial lymph node	Absent	Negative	N/A	Farm 1
TB 6987	83750237	Prescapsular lymph node	Absent	Negative	N/A	Farm 1
TB 6988	83750239	Prescapsular lymph node	Absent	Negative	N/A	Farm 1
TB 6889	83750219	Prescapsular lymph node	Absent	Negative	N/A	Farm 1
TB 6990	83750221	Mesenteric lymph node	Absent	Negative	N/A	Farm 1
TB 6991	83750224	Mesenteric lymph node	Absent	Negative	N/A	Farm 1
TB 6992	83750226	Prescapsular lymph node	Absent	Negative	N/A	Farm 1
TB 6993	83750227	Prescapsular lymph node	Absent	Negative	N/A	Farm 1
TB 6994	83750228	Prescapsular lymph node	Absent	Negative	N/A	Farm 1
TB 6995	83750229	Prescapsular lymph node	Absent	Negative	N/A	Farm 1
TB 6996	83750235	Mesenteric lymph node	Absent	Negative	N/A	Farm 1
TB 6997	83750236	Bronchial lymph node	Absent	Negative	N/A	Farm 1
TB 6998	83750240	Lung	Absent	Negative	N/A	Farm 1
TB 6999	83750242	Prescapsular lymph node	Absent	Negative	N/A	Farm 1
TB 7000	25545	Lung	Absent	Positive	*M. tuberculosis*	Farm 1
TB 7044	84750360	Mediastinal lymph node	Absent	Negative	N/A	Farm 2
TB 7045	84750366	Mesenteric lymph node	Absent	Negative	N/A	Farm 2
TB 7046	84750367	Prescapsular lymph node	Absent	Positive	*M. tuberculosis*	Farm 2
TB 7047	23738	Mediastinal lymph node	Absent	Positive	NTM species closely related to *M. moriokaense*	Farm 2
TB 7048	84750345	Mediastinal lymph node	Absent	Negative	N/A	Farm 2
TB 7049	84750359	Mediastinal lymph node	Absent	Negative	N/A	Farm 2
TB 7050	84750346	Bronchial lymph node	Absent	Negative	N/A	Farm 2
TB 7051	84750334	Bronchial lymph node	Absent	Negative	N/A	Farm 2
TB 7052	84750349	Bronchial lymph node	Absent	Negative	N/A	Farm 2

*NTM* Non-tuberculous mycobacterium, *N/A* Not applicable

**Fig. 5:**
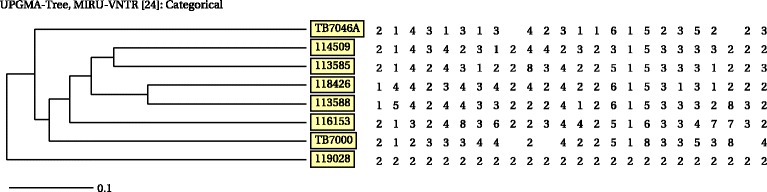
Phylogenetic tree based on a 24 variable number of tandem repeat (VNTR) data illustrating the genetic relationship of the cattle *M. tuberculosis* strains in comparison to a selection of human *M. tuberculosis* strains in the National Institute of Communicable Diseases (NICD) human database for strains identical at 5–10 VNTR loci originating from different regions in South Africa. The human isolates originated from the KwaZulu-Natal and North West Provinces of South Africa

We compared the cattle *M. tuberculosis* strains VNTR profiles (Table 2) to those of humans available in the database at the National Institute of Communicable Disease (NICD) and found no match to any of the human VNTR profiles from the Eastern Cape Province as well as other provinces. According to the phylogenetic tree (Fig. 5), which was constructed using VNTR data from cattle and human strains identical at 5–10 VNTR loci, no genetic relatedness amongst the strains could be established.

**Table 2:** Cattle identification, sample types and 24-locus variable number of tandem repeat profiles of *M. tuberculosis* strains isolated in the Eastern Province of South Africa

Locus name	MIRU 04	MIRU 26	MIRU 40	MIRU 10	MIRU 16	MIRU 31	VNTR 42	VNTR 43	ETR A	VNTR 47	VNTR 52	VNTR 53	QUB 11b	VNTR 1955	QUB 26	MIRU 02	MIRU 23	MIRU 39	MIRU 20	MIRU 24	MIRU 27	VNTR 46	VNTR 48	VNTR 49	Animal ID	Sample type	Farm
Copy number	3	8	3	3	4	5	1	2	n/a	2	4	n/a	2	4	8	2	5	3	n/a	1	3	3	2	3	25545 (TB 7000)	Lung	Farm 1
	3	5	1	3	1	5	1	4	2	1	3	2	4	3	n/a	2	6	2	n/a	1	2	3	1	3	84750367 (TB 7046A)	Prescapsular LN	Farm 2

*n/a* not available (PCR was not successful), *LN* Lymph node

## Discussion

In this study, we have isolated and identified *M. tuberculosis* from two cattle originating from two epidemiologically unrelated farms in the Eastern Cape Province of South Africa. The use of genomic regions of difference (i.e. RD4; RD9 and RD12) as described by Warren and co-workers [23] was useful in the correct identification of the isolates since both bovine and human tuberculosis have been previously detected in the country [17; 19]. According to Biet and co-workers, the route of TB transmission can be determined by observing the pattern or location where lesions are found in slaughtered animals [24]. Nonetheless, in the current report, no visible tuberculous lesions were observed in the cattle samples processed in both cases, and these observations corroborated previous findings from other studies [6, 10, 25]. Indeed, the severity of pathology in cattle infected with *M. tuberculosis* has been found to be lower compared to that of cattle infected with *M. bovis* [3], which explains the absence of lesions in these cases. In addition, cattle were also previously thought to be quite resistant to *M. tuberculosis* infection [24]. Our findings further supports a previous report which highlighted the importance of collecting samples from tissues prone to tuberculosis infection even where there are no visible lesions from animals that reacted positive on tuberculin skin test as *M. tuberculosis* may be isolated from such samples [23]. Isolation of *M. tuberculosis* species from a lung and a prescapsular lymph node from the two cases respectively may suggest erogenous transmission [26]. We suspect humans suffering from active tuberculosis as possible sources of infection through close contact with the cattle. This may not be surprising since our country has a high incidence of tuberculosis infection in humans [18]. Ideally, to unequivocally confirm or link humans as sources of the *M. tuberculosis* infections, the disease status of humans on the respective farms should be investigated. Unfortunately, this important action could not be carried out at the time, a move that would have embraced the concept of ‘One Health’. However, the genotypes from the cattle strains of *M. tuberculosis* were compared to those from local and national human data bases at the National Institute of Communicable Diseases (NICD) and no genetic relatedness amongst the strains could be established. These findings suggest lack of epidemiological relationship between the cattle strains and those available in the databases. Nonetheless, the VNTR profiles identified in the current study will form the basis for creating a genotyping database for animal *M. tuberculosis* strains for future epidemiological studies in South Africa.

Although *M. tuberculosis* infections is a problem in humans in our country, the prevalence in cattle is not known since no prevalence studies have been published so far. Additionally, for all cases of bovine tuberculosis confirmations done by the Tuberculosis laboratory of ARC-Onderstepoort Veterinary Research, which is a national bovine tuberculosis laboratory for surveillance and control purposes, only *M. bovis* was detected from all other cattle cases. To the best of our knowledge, our findings represent the first reported cases of *M. tuberculosis* infections in cattle in South Africa, however, cases of *M. tuberculosis* in captive wildlife have been reported. In these reports, humans were suspected as possible sources of infections [27, 28]. Both incidences of *M. tuberculosis* infections in cattle and in captive wildlife species pose a threat of spill back to humans since these animal species and humans are in most cases in close contact and direct transmission may occur. In a study conducted in Ethiopia, although investigators could not identify any transmission of *M. tuberculosis* between human and cattle, they suspected that the local practice of chewing tobacco and spitting into the mouths of cattle with the purpose of enhancing animal performance may have played a role in the transmission of *M. tuberculosis* to cattle, suggesting transmission by ingestion [3, 14]. In addition, they speculated that cattle became infected through aerosol inhalation from human while brought into the farmer’s house at night [3].

We have also isolated non-tuberculous Mycobacterium (NTM) species, *Mycobacterium nonchromogenicum,* from a prescapsular and mesenteric lymph node of a cow on farm 1, and an NTM species closely related to *Mycobacterium moriokaense* from a mediastinal lymph node of a cow on the other farm. Similar to isolation of *M. tuberculosis*, the NTM species were also detected from samples without visible lesions and therefore their clinical significance could not be determined in this study. However, their isolation from cattle which were suspects or reacted positively upon tuberculin skin testing is not surprising since some NTM species are known to cross react against *M. bovis* antigens in tuberculin based TB tests. Indeed, Gcebe and co-workers found shared antigens between *M. bovis* and *M. nonchromogenicum* using comparative genomics and proteomics approaches [29]. Additionally, in their follow up study to evaluate the immunogenicity of a purified protein derivative (PPD) prepared from *M. nonchromogenicum,* they found that indeed *M. nonchromogenicum* does elicit an immune response which is cross reactive against *M. bovis* antigens using routine diagnostic samples from cattle and African buffaloes (*Syncerus caffer*) in a modified gamma interferon assay (Gcebe et al., unpublished). Furthermore, *M. nonchromogenicum* was previously found to be amongst the four prevalent species in South African cattle, buffaloes and their environments [30].

## Conclusion

The cases described in this paper highlight *M. tuberculosis* as a zoonotic threat and a significant veterinary and an ongoing public health challenge in our country. The study reinforces the need for collaborative efforts between the veterinary and the public health services to ensure that detailed epidemiological investigations are carried out in cases such as these.

## Methods

### Tissue samples and *Mycobacterium* species cultivation

In 2008, tissue samples from cattle originating from two epidemiologically unrelated commercial farms (farm 1; *n* = 20, from 19 animals, and farm 2; *n* = 9, from 9 animals) located in the E.C Province of S.A were submitted to the Tuberculosis Laboratory at the Agricultural Research Council-Onderstepoort Veterinary Research (ARC-OVR) for routine *Mycobacterium* species isolation. *M. bovis* infection was previously detected from cattle on farm 1 [17], however, the bovine tuberculosis status on farm 2 was unknown. Routine submissions at the Tuberculosis laboratory of the ARC-OVR form part of the State Veterinary Service’s strategy for confirming tuberculosis infections in either skin test positive/suspect reactor or slaughter cattle with suspect tuberculous lesions. In these two cases, suspect or positive reactor cattle were slaughtered and sampled subsequent to a comparative intradermal tuberculin testing using bovine and avian PPD (Prionics, Lelystad) conducted by a local State Veterinarian. No information was provided on the sample submission form, which accompanied the samples, regarding the total number of animals, tested using the intradermal skin test. Bovine tuberculosis is a controlled disease in South Africa according to the animal disease legislation act 35 of 1984. Handling of animals during slaughter and sampling was done by professional Animal Health Technicians and State Veterinarians according to their procedures, and no animal ethics approval was required. Predilection sites prone to TB infection were sampled (Table 1). In the laboratory, tissue samples to be processed for mycobacterial isolation were carefully examined macroscopically for formation of suspected tuberculous-like lesions (visible). The presence or absence of suspect TB-like lesion was recorded for each sample (Table 1). The samples were processed and cultured according to standard laboratory procedures. Briefly, approximately 5 g of tissue samples were cut into small pieces and covered with 100 ml of sterile distilled water in a biohazard cabinet (Esco Class II BSC; Labotec, SA). The samples were homogenized using the Ultra-Turrax® homogenizer (Separation Scientific, SA). Seven millilitres of the homogenates were poured into two separate 15 ml falcon tubes, and decontaminated with 7 ml of 2% HCL and 7 ml of 4% NaOH for 10 min. Following centrifugation at 2360 g for 10 min (Labofuge 400, Haraeus Instruments), the supernatant was discarded and sterile distilled water was added to the pellet. The centrifugation step was repeated and the supernatant was discarded. The pellet was inoculated onto Löwenstein-Jensen (L-J) media slopes supplemented with glycerol and pyruvate; which are known to promote growth of mycobacteria; and incubated at 37 °C for up to 10 weeks with weekly monitoring.

### Ziehl Neelsen staining

Bacterial growth observed during monitoring was subjected to Ziehl Neelsen (ZN) staining to check for acid-fast bacteria (AFB). Bacterial smears were prepared from colonies on a microscopic slide. The stained slides were viewed under a microscope for AFB.

### Preparation of mycobacterial cell lysate

Cell lysates of acid-fast bacteria were prepared by picking up larger individual colonies or several small colonies from the L-J media. Bacteria were suspended in 100 μl sterile distilled water. The suspensions were boiled at 100 °C for 25 min, cooled down to room temperature and then transferred either 4 °C (short term storage) or −20 °C for long term storage [31].

### Identification of *Mycobacterium tuberculosis* complex species by PCR

Acid-fast isolates were subjected to amplification by PCR to identify MTBC bacteria using primers that target a sequence encoding the MPB70 antigen [32]. For each AFB isolate, a 50 ul PCR reaction was prepared containing the following components: 25 μl Ultra-pure water; 5 μl of 10 X reaction buffer (Separation Scientific), 3 μl of 25 mM MgCl_2_ (Separation Scientific); 2.5 μl of 1 mM dNTP (Inqaba Biotechlonogies Industries); 2 μl of 20 pMol/ul TB1A forward primer (5′ GAACAATCCGGAGTTGACAA 3′); 2 μl of 20 pMol/ul TB1B reverse primer (5′ AGCACGCTGTCAATCATGTA 3′) (Inqaba Biotechlonogies Industries) and 0.5 μl of SuperTherm Taq polymerase (Separation Scientific). The master mix was well mixed while avoiding bubbles and 39 μl of it was aliquoted into pre labelled micro centrifuge tubes as well as 10 μl of the template DNA (bacterial lysate). The PCR cycling parameters were as follows: initial denaturation at 94 °C for 5 min, next, each cycle consisted of denaturation at 94 °C for 30 s; annealing at 64 °C for 30 s, elongation at 72 °C for 2 min (40 cycles) and holding at 4 °C. PCR amplification was carried out using an Eppendorf AG 22331 Hamburg thermo cycler (Merck). The PCR products were separated on a 1.5% agarose gel stained with 20 μl ethidium bromide (10 μg/ml) and run at 80 V for 3 h. A 100 bp ladder (Inqaba Biotechnical Industries) was included. A product size of 372 bp was expected for MTBC species.

### Identification of *Mycobacterium tuberculosis* by PCR

For each MTBC isolate, a PCR reaction contained 5 μl 10X–buffer (Separation Scientific); 2 μl 25 mM MgCl_2_ (Separation Scientific); 4 μl of 10 mM dNTP (Inqaba Biotechnologies); 0.5 μl of 50 pmol/μl of either RD4; RD9 and RD12 primers (Inqaba Biotechnologies) (RD 4 forward = 5′-ATGTGCGAGCTGAGCGATG-3′; RD4 internal = 5′-TGTACTATGCTGACCCATGCG-3′ and RD4 reverse = 5′-AAAGGAGCACCATCGTCCAC-3; RD9 forward: 5′-CAAGTTGCCGTTTCGAGCC-3′; RD9 internal: 5′-CAATGTTTGTTGCGCTGC-3′; RD9 reverse: 5’GCTACCCTCGACCAAGTGTT-3′; RD12 forward: 5′-GGGAGCCCAGCATTTACCTC-3′; RD12 internal: 5’GTGTTGCGGGAATTACTCGG-3′; RD12 reverse: 5′-AGCAGGAGCGGTTGGATATTC-3′); 0.4 μl SuperTherm DNA polymerase (Separation Scientific) and 2 μl DNA template. The mixture was made up to 25 μl with sterile distilled water. PCR amplifications were carried out for individual primer sets as follows: initial denaturation at 94 °C for 15 min, followed by 45 cycles at 94 °C for 1 min; annealing at 62 °C for 1 min; elongation at 72 °C for 1 min. After the last cycle, the samples were incubated at 72 °C for 10 min. PCR amplification was carried out using an Eppendorf AG 22331 Hamburg thermo cycler (Merck). The PCR products were separated on a 2% agarose gel stained with 20 μl ethidium bromide (10 μg/ml) and run at 80 V for 3 h. A 100 bp ladder (Inqaba Biotechnical Industries) was included. Product sizes of 172 bp, 235 bp and 369 bp for RD4, RD9 and RD12 respectively, were expected [23].

### Identification of non-tuberculous mycobacteria

Non-tuberculous mycobacteria (NTM) were identified by PCR and sequence analysis of the 577 bp of the Mycobacterium species 16S rDNA. PCR targeting a 577 bp fragment of mycobacterial 16S rDNA was performed using the primers: 16S–F (5′-AGA GTT TGA TCM TGG CTCAG-3′) and 16S–R (5′-GCG ACA AAC CAC CTA AGA G-3′). Culture suspensions were used as DNA template in a 25 μl PCR mixture containing 12.9 μl deionized water, 2.5 μl of 10X PCR buffer (160 mM) (Tris Cl, KCl, (NH_4_)_2_SO4), 2 μl MgCl_2_ (25 mM), 1 μl dNTPs (10 mM), 0.1 μl Taq polymerase (Qiagen Hotstar Taq, Whitehead Scientific, South Africa), 5 μl of 5× Q-solution, 1 μl of each forward and reverse primers (50 pmol) and 1–2 μl DNA template. The PCR cycling parameters were as follows: initial denaturation at 95 °C for 15 min, followed by 35 cycles of denaturation at 95 °C for 30 s, annealing at 60 °C for 30 s and elongation at 72 °C for 30 s and a final extension at 72 °C for 10 min. The amplicons were sent to Inqaba Biotechnologies, South Africa for sequencing of the forward 16S rDNA strands using an ABI sequencer. Sequences were edited manually and pairwise alignments undertaken using the BioEdit Sequence alignment editor (version 7.1.9) and Molecular Evolutionary Genetics Analysis (MEGA) platform [30]. The sequences were then analyzed on the NCBI BLAST platform for species identification by mega blast [25].

### Genotyping

*M. tuberculosis* isolates were genotyped using a standardized 24 loci variable number of tandem repeat (VNTR) as previously described [33]. The loci were amplified individually as previously described [34, 35]. VNTR typing PCR was performed in a 20 μl reaction containing 2 μl of DNA, 10 μl of the Qiagen master mix, 7 μl of DNA free water and 0.5 μl of each 20 pM primer. The cycling parameters were as follows: initial denaturation at 94 °C for 5 min, followed by 40 cycles of denaturation at 94 °C for 1 min, annealing at 62 °C for 1 min, elongation at 72 °C for 1.5 min and a final elongation step at 72 °C for 10 min. PCR was carried out using an Eppendorf AG 22331 Hamburg thermo cycler (Merck). The PCR products were separated on a 2% agarose gel stained with 20 μl ethidium bromide (10 μg/ml) and run at 80 V for 3 h. A 100 bp ladder (Inqaba Biotechnical Industries) was included and used to estimate the sizes of the resulting PCR products. The VNTR profiles were recorded as a series of numbers corresponding to the number of alleles at each locus (Table 2) and were compared to human VNTR profile database (from the Eastern Cape Province and other provinces) at the National Institute of Communicable Diseases. From the entire database, we selected those human *M. tuberculosis* strains, which were identical to the cattle strains at 5–10 VNTR loci for construction of a dendrogram [36]. The dendrogramme was constructed using the MIRU-VNTR*plus* database (www.miru-vntrplus.org/). The results were entered into the database as numerical codes corresponding to the number of alleles at each locus. The categorical coefficient was used to calculate the distance matrix and the dendrogramme was constructed using the Unweighted Pair Group Method with Arithmetic averages (UPGMA) algorithm.
